# Supplementary material for: A novel and effective method for solving the router nodes placement in wireless mesh networks using reinforcement learning
Source: PLoS One. 2024 Apr 10;19(4):e0301073. doi: 10.1371/journal.pone.0301073 (PMC11006202; doi:10.1371/journal.pone.0301073)
Supplement: Attachment — Submitted filename: Response_to_Reviewers.pdf [file pone.0301073.s002.pdf]

Dear Editors and Reviewers,

On behalf of the authors, I would like to thank you for reviewing our manuscript. The comments of the Editors and Reviewers are valuable and exceptionally supportive for revising and improving the manuscript. We have carefully considered the comments and revised them accordingly. The revised content has been highlighted specifically as follows:

Journal requirements:

When submitting your revision, we need you to address these additional requirements.
1. Please ensure that your manuscript meets PLOS ONE's style requirements, including those for file naming. The PLOS ONE style templates can be found at https://journals.plos.org/plosone/s/file?id=wjVg/PLOSOne_formatting_sample_main_body.pdf and https://journals.plos.org/plosone/s/file?id=ba62/PLOSOne_formatting_sample_title_authors_affiliations.pdf

Author response: Thank you for the comments of the Editorial Board. We have checked and edited the manuscript to it meets PLOS ONE's style requirements.

2. Please note that PLOS ONE has specific guidelines on code sharing for submissions in which author-generated code underpins the findings in the manuscript. In these cases, all author-generated code must be made available without restrictions upon publication of the work. Please review our guidelines at https://journals.plos.org/plosone/s/materials-and-software-sharing#loc-sharing-code and ensure that your code is shared in a way that follows best practice and facilitates reproducibility and reuse.

3. In your Data Availability statement, you have not specified where the minimal data set underlying the results described in your manuscript can be found. PLOS defines a study's minimal data set as the underlying data used to reach the conclusions drawn in the manuscript and any additional data required to replicate the reported study findings in their entirety. All PLOS journals require that the minimal data set be made fully available. For more information about our data policy, please see http://journals.plos.org/plosone/s/data-availability.

Author response: Thank you for the comments of the Editorial Board. We have provided the minimal data set in the Supporting Information files.

4. PLOS requires an ORCID iD for the corresponding author in Editorial Manager on papers submitted after December 6th, 2016. Please ensure that you have an ORCID iD and that it is validated in Editorial Manager. To do this, go to 'Update my Information' (in the upper left-hand corner of the main menu), and click on the Fetch/Validate link next to the ORCID field. This will take you to the ORCID site and allow you to create a new iD or authenticate a pre-existing iD in Editorial Manager. Please see the following video for instructions on linking an ORCID iD to your Editorial Manager account: https://www.youtube.com/watch?v=_xcclfuvtxQ".

Author response: Thank you for the comments of the Editorial Board. We have updated the ORCID for all authors.

5. We note that Figure 1, 2, and 3 in your submission contain copyrighted images. All PLOS content is published under the Creative Commons Attribution License (CC BY 4.0), which means that the manuscript, images, and Supporting Information files will be freely available online, and any third party is permitted to access, download, copy, distribute, and use these materials in any way, even commercially, with proper attribution. For more information, see our copyright guidelines: http://journals.plos.org/plosone/s/licenses-and-copyright.

Author response: Thank you for the comments of the Editorial Board. These images were created by us. These may contain copyrighted icons. We have edited these images as follows:

- Figures 1 and 2 were drawn using Microsoft Office 365 Words. We have edited these two figures by removing icons.
- Figure 3 was drawn using MATLAB, we have edited this figure.

6. Please include captions for your Supporting Information files at the end of your manuscript, and update any in-text citations to match accordingly. Please see our Supporting Information guidelines for more information: http://journals.plos.org/plosone/s/supporting-information.

Author response: Thank you for the comments of the Editorial Board. We have added supporting information to the manuscript.

7. We are unable to open your Supporting Information file [LateXSourceFile.zip]. Please kindly revise as necessary and re-upload.

Author response: Thank you for the comments of the Editorial Board. We have edited and re-uploaded this file.

Reviewers' comments:

Reviewer's Responses to Questions

Comments to the Author

Reviewer #1:

1. Usage of English should be improved. Especially in the abstract and Introduction section, the authors should provide more details on the motivation behind the work.

Author response: Thank you for your valuable comments. We sincerely apologize for the errors in the writing of the manuscript. We have checked and corrected all errors in the manuscript. The motivation for this work has been added to the Abstract.

2. Figures should be included in the main text. Further, the quality of figure is not good. High defination images should be used.

Author response: Thank you for your valuable comments. According to the PLOS ONE guidelines, all images must be submitted as separate files and not included in the main document. Therefore, we retained only captions at the locations of the Figures. All images were submitted separately and automatically included at the end of the manuscript. In addition, we have added supporting information sections describing the figures and datasets. For the quality of the figures, we have made edits to improve the quality of the images.

3. The author should justify in the introduction section that how the proposed work is unique and superior than the following literature:

Author response: Thank you for introducing new articles with interesting content. Among these, article (d) is relevant to our work. We have carefully surveyed this work ([35]) and have updated the introduction (section I, page 2).

We have added a paragraph in the introduction to present the differences and superiority of the proposed method compared to the method using optimization algorithms, as in (d) and some previous studies (pages 2 and 3).

[a] Ren, Haoxing, and Matthew Fojtik. "Nvcell: Standard cell layout in advanced technology nodes with reinforcement learning." In 2021 58th ACM/IEEE Design Automation Conference (DAC), pp. 1291-1294. IEEE, 2021.

[b] Sharma, Anamika, and Siddhartha Chauhan. "A distributed reinforcement learning based sensor node scheduling algorithm for coverage and connectivity maintenance in wireless sensor network." Wireless Networks 26, no. 6 (2020): 4411-4429.

[c] Ouamri, Mohamed Amine, Gordana Barb, Daljeet Singh, and Florin Alexa. "Load balancing optimization in software-defined wide area networking (SD-WAN) using deep reinforcement learning." In 2022 International Symposium on Electronics and Telecommunications (ISETC), pp. 1-6. IEEE, 2022.

[d] Taleb, Sylia Mekhmoukh, Yassine Meraihi, Seyedali Mirjalili, Dalila Acheli, Amar Ramdane-Cherif, and Asma Benmessaoud Gabis. "Mesh Router Nodes Placement for Wireless Mesh Networks Based on an Enhanced Moth–Flame Optimization Algorithm." Mobile Networks and Applications (2023): 1-24.

[e] Ouamri, Mohamed Amine, Mohamed Azni, Daljeet Singh, Waleed Almughalles, and Mohammed Saleh Ali Muthanna. "Request delay and survivability optimization for software defined-wide area networking (SD-WAN) using multi-agent deep reinforcement learning." Transactions on Emerging Telecommunications Technologies (2023): e4776.

4. Required citation to the literature should be given for mathematical expressions in the paper. For example, for Connected router ratio and Connected client ratio.

Author response: Thank you for your valuable comments. The mathematical equations that we referred to in previous studies have been cited.

5. A comparison of computational complexity should be presented to fully justify the proposed algorithm.

Author response: Thank you for your valuable comments. We have added a paragraph after Algorithm 1 (pages 8 and 9) to carefully analyze the computational complexity of the proposed algorithm.

The computational complexity of Algorithm 1 depends mainly on the iteration in Step (2), the number of possible actions in Step (4), and the algorithm for updating the Q value in Step (5). $Q(r_i, s_t, a_{t,j})$ is updated using Eq. (11), where the greatest complexity is the calculation of $RW(r_i, s_t, a_t)$ according to (10). $RW(r_i, s_t, a_t)$ contains two metrics, *CRR* and *CCR*, which are defined by (1) and (5), respectively. To determine *CRR*, we employed a breadth-first search algorithm on a network of m vertices, which is the number of mesh routers. Therefore, the computational complexity was $O(m^2)$. The *CCR* is calculated using two nested loops of sizes m and n, where n is the number of mesh clients. Therefore, the complexity was $O(m \times n)$. Because n is always greater than m in a WMN, the computational complexity of $RW(r_i, s_t, a_t)$ is $O(m \times n)$. Consequently, the computational complexity of Algorithm 1 is $O(I \times |A| \times m \times n)$, where I is the number of iterations and $|A|$ is the number of possible actions.

The computational complexity of Algorithm 1 is greater than that of the algorithms solving the RNP problem using GA [38], PSO [23], and WOA [39] which we compare in the following section. However, because its computing complexity is a polynomial function, it can be implemented in

practice. Furthermore, because the algorithms for solving the RNP problem are run offline, the polynomial complexity is acceptable.

Reviewer #2:

The paper discusses the challenge of Router Node Placement (RNP) in wireless mesh networks, a known NP-hard problem. Traditional algorithms cannot efficiently solve this problem, so the paper introduces a novel approach using reinforcement learning. They model RNP as a reinforcement learning problem with network components representing the environment, routers, coordinate adjustment, and connectivity. This study is the first to apply reinforcement learning to RNP. Experimental results demonstrate that their method surpasses recent methods in terms of network coverage and connectivity.

1. Although the authors have discussed some essential background, I still do not well catch the main novelty directly. The challenges, methodology, and improvements need to be better clarified, both from research and application aspects, in the Introduction section.

Author response: Thank you for your valuable comments. We have revised and expanded the introduction to make the novel contributions of this paper more evident (pages 2 and 3).

2. The novelty of this paper needs to be highlighted.

Author response: Thank you for your valuable comments. We have revised the introduction to make the novel contributions of this paper more evident. The main contributions of this paper are summarized as follows:

(i) We propose a novel and effective method for solving the RNP problem by using RL. The RNP problem is modeled as an RL model, with the environment, agent, action, and reward representing the network system, routers, coordinate adjustment, and connectivity, respectively, of the RNP problem. To the best of our knowledge, this is the first study to apply reinforcement learning to the RNP problem.

(ii) We compared and evaluated the performance of the RNP problem-solving method using the heuristic optimization algorithms and the RL method.

3. The motivation to the problem need to be discussed in the abstract. Why authors are considering RL instead of Approximation or Heuristics.

Author response: Thank you for your valuable comments. We have edited the abstract to present the motivation for this work more clearly.

4. Figures should keep in suitable location.

Author response: Thank you for your valuable comments. According to the PLOS ONE guidelines, all images must be submitted as separate files and not included in the main document. Therefore, we retained only captions at the locations of the Figures. All images were submitted separately and automatically included at the end of the manuscript. In addition, we have added supporting information sections describing the figures and datasets.

5. There are typos, grammatical errors, punctuation errors and other mistakes. Some sentences are not clear. For example: "In addition, there is at least one the router of the internet".

Author response: Thank you for your valuable comments. We have thoroughly reviewed and edited all drafting errors in the manuscript. Some unclear sentences have been rewritten accordingly.

6. What protocol is used to communicate when two routers are within the range of each other.

Author response: Thank you for your valuable comments. When two nodes are within the range of each other, the IEEE 802.11 protocol is used for the communication between them. The routing protocol used was Destination-Sequenced Distance-Vector Routing (DSDV).

7. Authors have to clarify whether they are comparing the proposed algorithm with the approximation algorithms or Heuristic algorithms.

Author response: Thank you for your valuable comments. The proposed algorithm was compared with heuristic algorithms GA, WOA, and PSO. We have corrected the presentation of the simulation results.

8. GA, PSO and WoA are not approximation algorithms.

Author response: Thank you for your valuable comments. We edited the manuscript accordingly. GA, PSO, and WOA are heuristic algorithms.

9. The authors have to consider the dynamic algorithms instead of static algorithms

Author response: Thank you for your valuable comments. In the scope of this paper, we focus on the static RNP problem because it is suitable for reinforcement learning models. In addition, the static RNP problem has many practical applications and has recently attracted the attention of many research groups. The application of reinforcement learning to solving the dynamic RNP problem is an interesting and practically meaningful research topic. We plan to investigate this topic in the near future. We have updated this content in the conclusion section.

10. The proposed algorithm is RL type, it is suggested that the proposed algorithm need to be compared with the RL algorithms only instead of comparing it with heuristic algorithms.

Author response: Thank you for your valuable comments. To the best of our knowledge, this is the first study to applies reinforcement learning to solve the RNP problem. We did not find any comparable studies for comparison. Therefore, we compared the proposed algorithm with heuristic algorithms.

11. Perform the comparative analysis with the latest works based on RL related placement problems.

Author response: Thank you for introducing these articles with many valuable contributions. We have carefully surveyed these papers and updated paper B (ref [35]) to the related work section (Introduction section). Paper A studied the application of deep reinforcement learning to wireless networks. However, the main focus was on optimizing the caching. In addition, our work focuses on the router node placement problem in a WMN. Therefore, we did not compare our results with those of Paper A.

A) "Deep reinforcement learning mechanism for deadline-aware cache placement in device-to-device mobile edge networks." Wireless Networks (2022): 1-20.

B) "Mesh Router Nodes Placement for Wireless Mesh Networks Based on an Enhanced Moth–Flame Optimization Algorithm." Mobile Networks and Applications, 1-24.

C) "An efficient mesh router nodes placement in wireless mesh networks based on moth-flame optimization algorithm". International Journal of Communication Systems, 36(8), e5468.

12. I suggest the authors include a subsection to show the complexity (Complexity of the proposed scheme), convergence, and optimality.

Author response: Thank you for your valuable comments. We have added a paragraph after Algorithm 1 (pages 8 and 9) to carefully analyze the computational complexity of the proposed algorithm.

The computational complexity of Algorithm 1 depends mainly on the iteration in Step (2), the number of possible actions in Step (4), and the algorithm for updating the Q value in Step (5). $Q(r_i, s_t, a_{t,j})$ is updated using Eq. (11), where the greatest complexity is the calculation of $RW(r_i, s_t, a_t)$ according to (10). $RW(r_i, s_t, a_t)$ contains two metrics, *CRR* and *CCR*, which are defined by (1) and (5), respectively. To determine *CRR*, we employed a breadth-first search algorithm on a network of m vertices, which is the number of mesh routers. Therefore, the computational complexity was $O(m^2)$. The *CCR* is calculated using two nested loops of sizes m and n, where n is the number of mesh clients. Therefore, the complexity was $O(m \times n)$. Because n is always greater than m in a WMN, the computational complexity of $RW(r_i, s_t, a_t)$ is $O(m \times n)$. Consequently, the

computational complexity of Algorithm 1 is $O(I \times |A| \times m \times n)$, where I is the number of iterations and $|A|$ is the number of possible actions.

The computational complexity of Algorithm 1 is greater than that of the algorithms solving the RNP problem using GA [38], PSO [23], and WOA [39] which we compare in the following section. However, because its computing complexity is a polynomial function, it can be implemented in practice. Furthermore, because the algorithms for solving the RNP problem are run offline, the polynomial complexity is acceptable.
