# Supplementary material for: A novel and effective method for solving the router nodes placement in wireless mesh networks using reinforcement learning
Source: PLoS One. 2024 Apr 10;19(4):e0301073. doi: 10.1371/journal.pone.0301073 (PMC11006202; doi:10.1371/journal.pone.0301073)
Supplement: Attachment — Submitted filename: ResponseToReviewers_r2.pdf [file pone.0301073.s004.pdf]

Dear Editors and Reviewers,

On behalf of the authors, I would like to thank you for reviewing and accepting most of the edits for the comments in the previous review. The comments of Editors and Reviewers are all valuable and exceptionally supportive for revising and improving the manuscript. We have carefully considered the remaining comments and have edited them. The revised content this time is on the track changes using LaTeX with color = orange and id = (r2).

Journal Requirements:

Please review your reference list to ensure that it is complete and correct. If you have cited papers that have been retracted, please include the rationale for doing so in the manuscript text, or remove these references and replace them with relevant current references. Any changes to the reference list should be mentioned in the rebuttal letter that accompanies your revised manuscript. If you need to cite a retracted article, indicate the article's retracted status in the References list and also include a citation and full reference for the retraction notice.

Author response: Thank you for the comments of the Editorial Board. We have reviewed the references. There are two changes in the reference list as follows:

(i) We have removed reference [36] because it overlaps with reference [27].

(ii) Two references [38] and [39] are added. These two references are introduced by reviewers. Through a careful survey of these works, we found that these are new research projects in the application of reinforcement learning in wireless networks, which are related to our article.

Reviewers' comments:

Reviewer's Responses to Questions

Comments to the Author

Reviewer #2: All of my concerns have been addressed. I congratulate the authors and All the best.

I recommend this work for possible publication. (Accepted)

The summer of the manuscript is:

The paper discusses the challenge of Router Node Placement (RNP) in wireless mesh networks, a known NP-hard problem. Traditional algorithms cannot efficiently solve this problem, so the paper introduces a novel approach using reinforcement learning. They model RNP as a reinforcement learning problem with network components representing the environment, routers, coordinate adjustment, and connectivity. This study is the first to apply reinforcement learning to RNP. Experimental results demonstrate that their method surpasses recent methods in terms of network coverage and connectivity.

Author response: Thank you very much for accepting the manuscript.

Reviewer #3: This paper presents a novel and effective method for solving the router nodes placement in wireless mesh networks using reinforcement learning.

1- The writing of paper should be improved seriously, equations should be cited and the content should be justify

Author response: Thank you for your valuable comments. We have reviewed and edited the manuscript, all equations have been fully cited except those proposed by us. The content is aligned to the left because it follows the template of PLOS ONE.

2- Please add the improvement of your method results in the abstract numerically.

Author response: Thank you for your valuable comments. We have added to the abstract the numerical value of increased network connectivity of the proposed method compared to the most recent methods.

3- Please compare your paper with the state-of-the art papers:

a. RLS2: An energy efficient reinforcement learning-based sleep scheduling for energy harvesting WBANs, by R Mohammadi…

b. DRDC: Deep reinforcement learning based duty cycle for energy harvesting body sensor node, by by R Mohammadi…

Thank you for introducing new articles of high scientific and practical significance. We investigated these works and have cited related work (ref. [38, 39]) on the topic of applications of reinforcement learning in wireless networks (page 6).

Since the problem in our work is different from the problem in those works (RLS2 and DRDC), we cannot compare our work with them. We can develop suitable research topics to compare with these works in the future.

4- you should use more approximation algorithms.

Author response: Thank you for your valuable comments. We used an additional approximation algorithm, which is the Multi-Verse Optimizer (MVO) algorithm to compare with the proposed algorithm. The experimental results have been updated in Figures 3 to 11 and the text analyzing the results of these figures.
