# Supplementary material for: A novel and effective method for solving the router nodes placement in wireless mesh networks using reinforcement learning
Source: PLoS One. 2024 Apr 10;19(4):e0301073. doi: 10.1371/journal.pone.0301073 (PMC11006202; doi:10.1371/journal.pone.0301073)
Supplement: Attachment — Submitted filename: review.docx [file pone.0301073.s003.docx]

This paper presents a novel and effective method for solving the router nodes placement in wireless mesh networks using reinforcement learning.

1- The writing of paper should be improved seriously, equations should be cited and the content should be justify
2- Please add the improvement of your method results in the abstract numerically.
3- Please compare your paper with the state-of-the art papers:
   a. [RLS2: An energy efficient reinforcement learning-based sleep scheduling for energy harvesting WBANs](https://scholar.google.com/citations?view_op=view_citation&hl=en&user=TVgCvw4AAAAJ&sortby=pubdate&citation_for_view=TVgCvw4AAAAJ:zA6iFVUQeVQC), by R Mohammadi…
   b. [DRDC: Deep reinforcement learning based duty cycle for energy harvesting body sensor node](https://scholar.google.com/citations?view_op=view_citation&hl=en&user=TVgCvw4AAAAJ&sortby=pubdate&citation_for_view=TVgCvw4AAAAJ:HoB7MX3m0LUC), by by R Mohammadi…
4- you should use more approximation algorithms.
